# In Situ Collection and Rapid Detection of Pathogenic Bacteria Using a Flexible SERS Platform Combined with a Portable Raman Spectrometer

**DOI:** 10.3390/ijms23137340

**Published:** 2022-07-01

**Authors:** Huimin Zhao, Dawei Zheng, Huiqin Wang, Taifeng Lin, Wei Liu, Xiaoli Wang, Wenjing Lu, Mengjia Liu, Wenbo Liu, Yumiao Zhang, Mengdong Liu, Ping Zhang

**Affiliations:** Faculty of Environment and Life, Beijing International Science and Technology Cooperation Base of Antivirus Drug, Beijing University of Technology, Beijing 100124, China; zhaohm@emails.bjut.edu.cn (H.Z.); zdv@bjut.edu.cn (D.Z.); wanghuiqin@bjut.edu.cn (H.W.); lintaifeng@bjut.edu.cn (T.L.); 13581830343@139.com (W.L.); wangxiaoli@bjut.edu.cn (X.W.); L2384130472@163.com (W.L.); liumengjia86@163.com (M.L.); liuwenbo2021@163.com (W.L.); zhangyumiao@emails.bjut.edu.cn (Y.Z.); 13522510559@163.com (M.L.)

**Keywords:** flexible SERS platform, pathogenic bacteria, rapid detection, in situ test

## Abstract

This study aims to develop a simple, sensitive, low-cost, environmentally friendly and flexible surface-enhanced Raman scattering (SERS) platform, combined with a portable Raman spectrometer, for the rapid and on-site SERS detection of bacteria. Commercial tobacco packaging paper (TPP) with little background interference was used as a loading medium that effectively adsorbed Au nanoparticles and provided sufficient “hot spots”. This Au-tobacco packaging paper (Au-TPP) substrate used as a flexible SERS platform can maximize sample collection by wiping irregular surfaces, and was successfully applied to the on-site and rapid detection of pathogenic bacteria. Raman fingerprints of pathogenic bacteria can be obtained by SERS detection of spiked pork using wipeable Au-TPP, which verifies its value in practical applications. The results collected by SERS were further verified by polymerase chain reaction (PCR) results. It showed several advantages in on-site SERS detection, including accurate discrimination, simple preparation, easy operation, good sensitivity, accuracy and reproducibility. This study indicates that the established flexible SERS platform has good practical applications in pathogenic bacterial identification and other rapid detections.

## 1. Introduction

In recent years, the detection of pathogenic bacteria has been of great significance to human health [1,2,3]. Until now, the conventional detection methods based on culture systems have been unable to meet the rapid requirements of bacteria [4]. Although some rapid methods such as polymerase chain reaction (PCR) and enzyme-linked immunosorbent assay (ELISA) are accurate and efficient, they still have limitations for on-site detection and rely on trained personnel [5,6,7,8]. Fortunately, surface-enhanced Raman scattering (SERS) technology has developed rapidly, which can not only build an ultrasensitive sensing platform with molecular recognition ability, but can also realize single-molecule detection [9,10]. SERS, as a powerful technique with sensitive, rapid and non-destructive features, can characterize the molecular structure of bacteria and enable rapid label-free detection [11,12,13,14,15,16,17]. In addition, there are a number of commercially available portable Raman spectrometers that can be easily used for field use [18,19].

Typical SERS substrates are based on rigid substrates such as silicon and glass, which are not suitable for sample collection by physical wiping [20,21,22,23]. An enhanced substrate with good flexibility can maximize sample collection by wiping irregular surfaces, enabling in situ detection and broadening the application of SERS technology [24,25,26,27,28,29]. Flexible SERS substrates have attracted extensive attention from researchers [30]. Various types of materials, including cellulose, polymer membranes, fabric, adhesive tape and biomaterials, have been investigated for use as flexible SERS substrates, which directly affect the effectiveness of SERS enhancements, the detection accuracy and sampling techniques [31]. Due to their ability to absorb nanoparticles and collect analytes, flexible SERS substrates are usually used with swab sampling [32]. The surface structure of flexible substrates helps to improve the uniform distribution of nanoparticles, resulting in high reproducibility. Kalachyova et al. obtained flexible SERS substrates with high SERS signal reliability and repeatability by exciting surface plasmon polarization waves on the periodical metal grating, supported by a thin polymer film, and demonstrated their potential in biological detection and recognition [33]. He et al. assembled silver dimers or directed aggregates with high SERS activity in polyvinyl alcohol nanofibers arranged in chains using electrostatic spinning technology, and successfully designed and synthesized an independent flexible SERS substrate, thus significantly improving the high sensitivity of SERS to 4-mercaptobenzoic acid (4-MBA) molecules with an enhancement factor of up to 10^9^ [34]. Several studies used 4-MBA as a probe molecule to evaluate the enhanced effects of fabricated and flexible SERS substrates. Moreover, 4-MBA is commonly used to form self-assembled monolayers (SAMs) on metal surfaces used for sensitive bioanalysis [35,36,37]. Various paper-based supporting media have been gradually taken into consideration for their characteristics of being flexible, low cost, and environmentally friendly [38,39,40,41,42]. Villa et al. deposited suspensions of various bacterial samples directly onto a filter paper-based SERS substrate for the rapid detection of bacteria [43]. Oliveira et al. presents office paper decorated with silver nanostars as an easy to fabricate and low-cost plasmonic substrate, with applications in trace analyte detection by SERS [44]. Torul et al. presents a paper membrane-based SERS platform for the determination of blood glucose levels using a nitrocellulose membrane as the substrate paper [45]. Importantly, flexible SERS substrates can be combined with portable Raman devices or even with smartphones to provide possible on-site detection [46]. However, the preparation of flexible SERS substrates still faces many difficulties that need to be improved in many aspects [47,48,49,50,51,52]. Firstly, the background interference caused by the supporting medium increases the difficulty of Raman detection and brings great challenges to the subsequent data processing [53,54]. Secondly, the sample collection and pre-treatment process before Raman tests need to be simplified and optimized to fit to the on-site detection [55,56]. Therefore, there is still a great demand to prepare enhanced substrates effectively, economically and practically.

Tobacco packaging paper (TPP) is a specially designed thin paper with high longitudinal tensile strength, air permeability, and good biocompatibility [57,58]. The Raman signal of TPP is very weak compared to other types of paper and fibers. More importantly, the surface of tobacco packaging paper has a hierarchical structure of a porous staggered arrangement, which provides a 3D structure and allows metal nanoparticles to be adsorbed and form enough “hot spots” for SERS detection [59,60]. Therefore, tobacco packaging paper has the potential to be used as a loading medium to prepare a flexible SERS platform for in situ sample collection and rapid Raman detection. Considering its excellent flexibility, a TPP-based SERS platform can easily collect analytes by simply wiping the irregular surface of the object being tested, such as for detecting bacterial contamination and pesticide pollution on fruit peels, vegetables and others. In addition, the TPP-based enhanced substrate is portable and can be easily cut into different shapes and sizes according to actual needs. 

Herein, the development of a simple, flexible, sensitive, low-cost, and environmentally friendly TPP-based enhanced substrate for rapid and on-site SERS detection is discussed. The gold nanoparticles (Au NPs) used as enhanced substrates were precipitated or centrifuged onto the surface of commercial tobacco packaging paper. The formation of “hot spots” was controlled by adjusting the amount of Au NP attachment. The prepared flexible SERS substrate was successfully applied to detect pathogenic bacterial contamination in commercial pork. The Raman characteristic fingerprints of pathogenic bacteria can be easily obtained and accurately distinguished. The established flexible SERS platform combined with a portable Raman spectrometer is suitable for large-scale and on-site market screening. This shows the feasibility of rapid and culture-free identification of pathogenic bacteria in food safety and clinical testing.

## 2. Results and Discussion

### 2.1. Preparation and Characterization of Au NPs and Au-TPP Substrates

Noble metal colloids are commonly used to enhance substrates for SERS detection due to their good enhancement effects and easy preparation [61,62]. However, nanoparticle aggregation is inescapable with a storage time of more than 3 months. Solid substrates prepared by electron beam lithography, thermal deposition and other methods can obtain highly reproducible spectral data, but the cost of the substrate is usually expensive, and the shortcomings of solid substrates include them being hard and fragile, which will reduce the collection efficiency of the samples [55,63,64].

**Figure 1:**
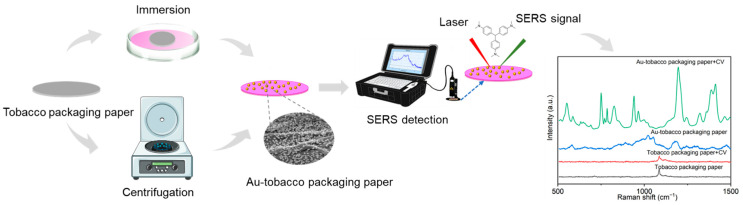
Schematic illustration of the preparation of the Au-TPP substrate and the SERS detection process.

**Figure 2:**
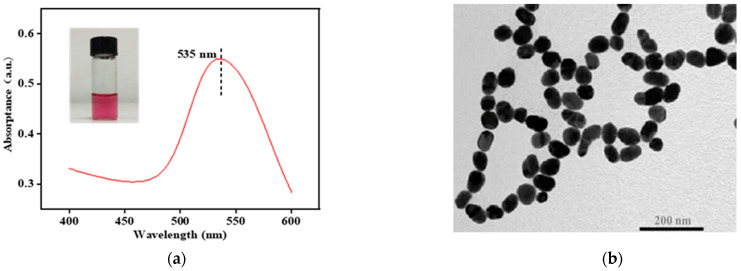
Characterization of the Au NP colloid. (**a**) The maximum single absorption peak of colloidal Au NPs is near 535 nm; (**b**) TEM image of Au NPs.

In this study, we synthesized a Au NP colloid and then used it to prepare the Au-tobacco packaging paper-based enhanced substrate (Au-TPP) by the immersion method and the centrifuge method (Figure 1). As shown in Figure 2a, the synthesized Au NP colloid is wine-red in color, and the maximum plasmon resonance absorption is near 535 nm. The Au NPs are approximately round with a smooth surface, and the size is relatively uniform without agglomeration in the transmission electron microscope (TEM) image (Figure 2b). Tobacco packaging paper (TPP) was chosen as a loading medium to prepare the flexible enhanced substrate due to its lower background signal interference and higher toughness.

### 2.2. Optimization of Preparation Conditions

**Figure 3:**
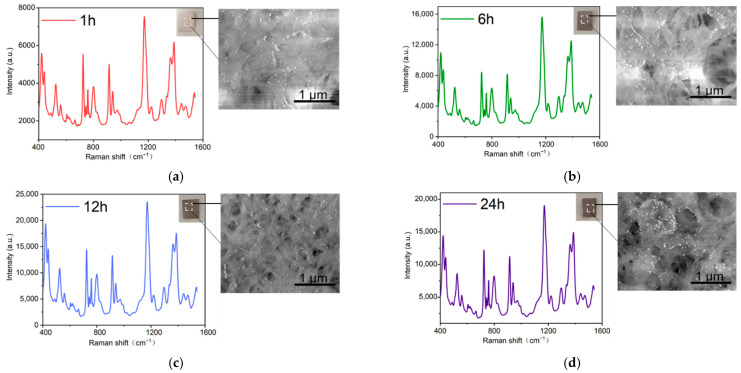

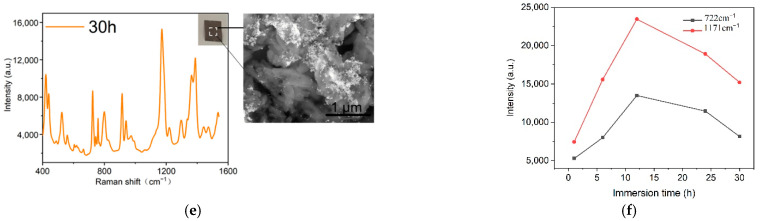
Optimum conditions for the preparation of the Au-TPP enhanced substrate by the immersion method. (**a**–**e**) SERS spectra of CV (10^−5^ M) using Au-TPP prepared using different immersion times, and related insets showing the SEM images of the Au-TPP; (**f**) the characteristic peak intensities of CV at 1171 cm^−1^ and 722 cm^−1^ changing with the immersion times.

The immersion method is one of the most common methods for preparing flexible substrates based on fiber paper [65,66]. The tobacco packaging paper was white before being immersed in colloidal Au NPs. With the prolongation of the immersion time, the color of the tobacco packaging paper gradually changed to dark brown, which means that more colloidal Au NPs were adsorbed into the tobacco packaging paper (Figure 3a–e). When the loading of colloidal Au NPs onto the surface of the tobacco packaging paper reaches the optimal value, the prepared Au-TPP substrate can generate more “hot spots”, thereby obtaining a stronger SERS spectral signal. The duration of the immersion time is the main factor that affects the adhesion of the Au NPs [65]. Therefore, the Au-TPP substrates were prepared with different immersion times (1 h, 6 h, 12 h, 24 h and 30 h), and crystal violet (CV) with a concentration of 10^−5^ M was selected as the probe molecule to examine the SERS enhancement abilities of the Au-TPP substrates under different immersion times. As shown in Figure 3f, as the immersion time increases, the CV enhancement effect of each Au-TPP substrate shows a trend of first increasing and then decreasing. When the immersion time is about 12 h, the Au NPs covering the SERS substrate of the tobacco packaging paper are in the best state and tend to be saturated. With the prolongation of the immersion time, the Au NPs covering the SERS substrate of the tobacco packaging paper were stacked and aggregated, so the SERS active “hot spots” decreased, and the enhancement effect decreased accordingly. Therefore, the Au-TPP substrate prepared by immersion for 12 h had the best enhancement effect. It can be observed from the scanning electron microscope (SEM) image that the blank tobacco packaging paper has a micron-level fiber structure, and the multilayer fibers are conducive to the adsorption of nanoparticles. It can be observed that the distribution of Au NPs is relatively uniform in the Au-TPP substrate immersed for 12 h, and there is not a large amount of aggregation.

**Figure 4:**
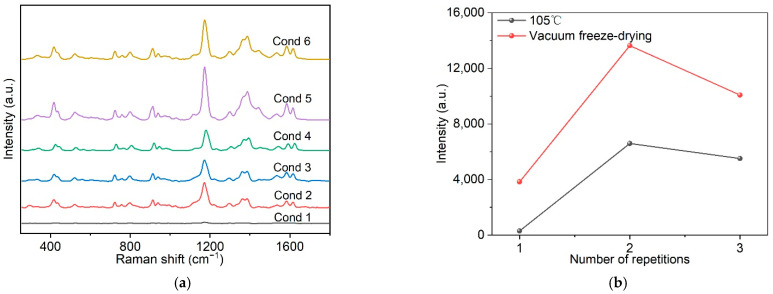

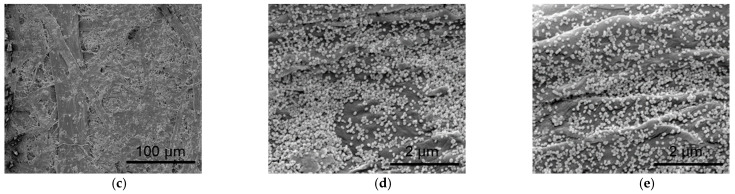
Optimum conditions for substrate preparation by centrifugation. (**a**) SERS spectra of CV (10^−5^ M) obtained from the Au-TPP substrates prepared under different conditions; (**b**) influence of the different drying methods on the peak intensities of 1171 cm^−1^; (**c**–**e**) SEM images: (**c**) blank tobacco packaging paper, Au-TPP substrate prepared by (**d**) Cond 2 and (**e**) Cond 5. Note: Cond 1 (2 mL): single centrifugation with 2 mL, drying at 105 °C; Cond 2 (4 mL): centrifugation with 2 mL, two times, 105 °C; Cond 3 (6 mL): centrifugation with 2 mL, three times, 105 °C; Cond 4 (2 mL): single centrifugation with 2 mL, vacuum freeze-drying; Cond 5 (4 mL): centrifugation with 2 mL, two times, vacuum freeze-drying; Cond 6 (6 mL): centrifugation with 2 mL, three times, vacuum freeze-drying.

Following on from the immersion method, the preparation of the flexible substrate was improved, and an active SERS substrate with good uniformity and high stability was successfully prepared using the centrifugal method. In the process of centrifugal preparation, it was found that different preparation conditions would affect the concentration, aggregation and distribution of metal nanoparticles on the surface of the substrate, thereby affecting the SERS enhancement effect, as shown in Figure 4a. The experiment explored the CV enhancement effect of a Au-TPP substrate prepared under different conditions by changing the times of colloidal Au NP bonding to the substrate, the amount of bonding with the substrate and the drying method. As shown in Figure 4b, and using the same drying method, the results show that as the amount of loaded colloidal Au NPs increases, the CV enhancement effect of the Au-TPP substrate first increases and then decreases. When comparing the drying methods, the SERS signal after vacuum freeze-drying is stronger than that of 105 °C drying. The reason for this may be that with the increase in the number of colloidal Au NPs attached to the substrate, agglomeration occurs, which reduces the active “hot spots” of SERS and the synergistic effect. The “hot spot” under vacuum freeze-drying is more active than when dried at 105 °C, and the generated SERS signal is stronger. It can be clearly observed from the SEM images that the surface of blank tobacco packaging paper has a hierarchical structure of a porous staggered arrangement (Figure 4c), and Au NPs are more uniformly distributed on the vacuum-dried Au-TPP substrate compared with drying at 105 °C (Figure 4d,e). Therefore, Cond 5 is the best condition to prepare the Au-TPP substrate.

The immersion method has disadvantages such as being time consuming, requiring large amounts of colloidal Au NPs, having high costs, and the inevitable coffee-ring effect between the Au NPs and the substrate. When compared with the immersion method, the centrifugation method is simpler and faster to prepare, and it only takes 10 min to prepare the Au-TPP substrate with a better enhancement effect. The preparation of SERS substrates by centrifugation also enables a controllable amount of Au NPs to be attached to the paper surface. According to the SEM image (Figure 4e), it is further proved that the centrifugal method can better make the Au NPs adhere to the surface of the substrate. A relatively uniform distribution of Au NPs can be observed on the surface of the Au-TPP substrate under Cond 5.

### 2.3. Uniformity, Reproducibility and Stability of the Au-TPP Substrate

**Figure 5:**
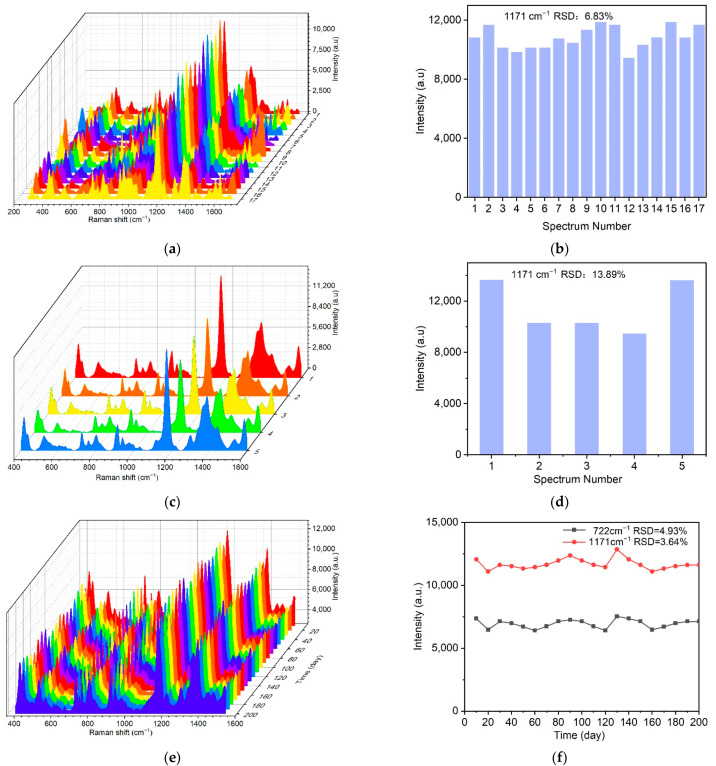
Uniformity, reproducibility and stability of the Au-TPP substrate. (**a**,**b**) Uniformity tests: (**a**) randomly select 17 different positions on the Au-TPP substrate to record the SERS signal of CV, (**b**) the RSD value of the corresponding signal calculated from the characteristic peak of 1171 cm^−1^; (**c**,**d**) reproducibility tests: (**c**) SERS spectra of CV on 5 batches of the Au-TPP substrate, (**d**) the RSD value of the corresponding signal calculated from the characteristic peak of 1171 cm^−1^; (**e**,**f**) stability tests: (**e**) SERS signal detection of CV using Au-TPP substrates stored for approximately 200 days, (**f**) peak intensities of 1171 cm^−1^ and 722 cm^−1^ recorded during different storage periods.

The SERS active substrate also requires the SERS signal to have good uniformity and reproducibility [38,67]. In order to evaluate the uniformity of the Raman signal distribution, 17 different positions on the Au-TPP substrate were selected and SERS measurements were performed under the same experimental conditions. The confocal point can move freely on the surface of the substrate, but all the spectral peak shapes are the same (Figure 5a). We calculated that the relative standard deviation (RSD) value of the characteristic peak intensity at 1171 cm^−1^ of the corresponding point of CV is 6.83% (Figure 5b), which suggests that the entire area of the SERS substrate shows good uniformity.

Under the same experimental conditions, the reproducibility of different batches of Au-TPP substrate was studied. Five batches of samples were randomly selected to detect CV. Shown in Figure 5c are the SERS signals obtained from the different batches of the Au-TPP substrates. For the different batches of the Au-TPP substrates, the SERS signal intensities are relatively consistent and present the same characteristic peak shape (Figure 5c). The relative standard deviation (RSD) value of the characteristic peak intensity at 1171 cm^−1^ is 13.89% (Figure 5d); therefore, the different batches of the Au-TPP substrates have good reproducibility.

The prepared Au-TPP substrates were sealed in opaque bags and stored at 4 °C for different times (0–200 d). The substrates were characterized every 10 d with CV as the probe molecule, and the enhancement effect was recorded to investigate their stability. With the increase in storage time, the SERS signals of the Au-TPP substrates were stable, and the RSDs of the characteristic peaks of CV at 722 cm^−1^ and 1171 cm^−1^ were both less than 10% (Figure 5e,f). This indicated that the Au-TPP substrates showed good stability after being stored for more than 3 months. By avoiding oxidation and sunlight, Au nanoparticles adsorbed on the paper fibers easily maintain more “hot spots” and provide stable SERS activity during the storage period, showing a long shelf-life.

### 2.4. Application in the Detection of Pathogenic Bacteria

**Figure 6:**
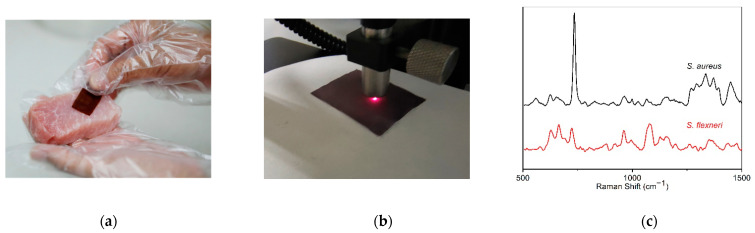

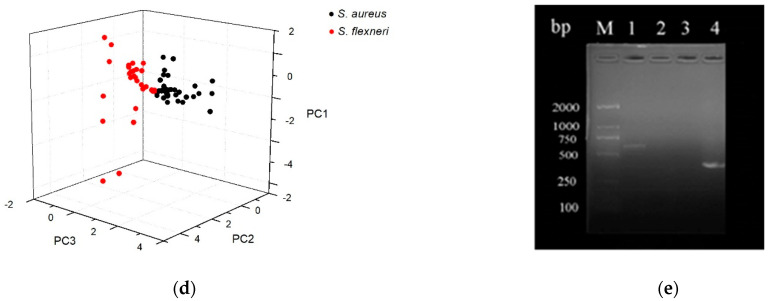
The detection of two pathogens based on the flexible SERS platform and PCR. (**a**) Sample collection process on spiked pork; (**b**) detection process with the portable Raman spectrometer; (**c**) SERS spectra of *S. flexneri* and *S. aureus* obtained from the Au-TPP substrates; (**d**) three-dimensional scatter plot of PC scores of *S. aureus* and *S. flexneri*; (**e**) PCR amplified *nuc* and *ipaH* gene expression. Note: M: PCR marker DL2000; 1: *S. aureus nuc* gene expression; 2: *S. aureus ipaH* gene expression; 3: *S. flexneri nuc* gene expression; 4: *S. flexneri ipaH* gene expression.

Due to its extremely high sensitivity and spectral signature of specific strains, SERS is considered a powerful tool for detecting pathogens in food safety and healthcare applications [17,68]. A Au-TPP flexible substrate can be used as a flexible SERS swab to collect pathogens present on various surfaces. For validation, we selected *Staphylococcus aureus* and *Shigella flexneri* as model pathogens, and collected bacteria by swabbing the surface of spiked pork (Figure 6a). Au-TPP substrate can be combined with portable Raman spectrometer. Raman fingerprints of pathogenic bacteria can be obtained by SERS detection of Au-TPP flexible substrate (Figure 6b). The SERS detection results proved that the use of a wipeable Au-TPP flexible substrate can effectively collect, identify and detect pathogens (Figure 6c). When compared with the background Raman signals of blank tobacco packaging paper and a Au-TPP substrate, it can be seen that the background Raman signals of blank tobacco packaging paper and the Au-TPP substrate are weaker, while the Raman signals of the two pathogenic bacteria are significantly enhanced by the Au-TPP substrate. The characteristic peaks of *S. aureus* are mainly 735 cm^−1^, 964 cm^−1^, 998 cm^−1^, 1026 cm^−1^, 1066 cm^−1^, 1162 cm^−1^, 1335 cm^−1^ and 1449 cm^−1^. The characteristic peaks of *S. flexneri* are mainly 626 cm^−1^, 665 cm^−1^, 723 cm^−1^, 875 cm^−1^, 920 cm^−1^, 961 cm^−1^, 996 cm^−1^, 1083 cm^−1^, 1156 cm^−1^, 1263 cm^−1^, 1351 cm^−1^, 1458 cm^−1^ and 1472 cm^−1^. It can be seen that the Au-TPP as a SERS substrate for pathogenic bacteria detection not only has the advantages of short detection time, simple operation, high sensitivity and specificity, but can also respond well to the Raman characteristic information of these two bacteria. 

The PCA method was used to analyze the SERS patterns (N ≥ 30) of two randomly selected strain samples. The three principal components PC1 (45.982%), PC2 (19.339%) and PC3 (12.425%) were obtained, and their cumulative contribution rate can reach 77.75%. Therefore, the discriminant classification model based on these three principal component factors is representative. The results are shown in Figure 6d, and the classification results of these two strains are 100% correct. Table 1 shows the linear discriminant classification results of the two pathogenic bacteria. The sensitivity and specificity are as high as 100%. Therefore, it is feasible to use Au-TPP as the SERS substrate to detect different types of pathogenic bacteria. 

The *nuc* gene encodes a thermostable nucleotidase produced by pathogenic staphylococci, which can realize the rapid identification of *S. aureus*. The *ipaH* gene is the most important virulence pathogenic gene in *Shigella*. The PCR results show that the *nuc* gene is expressed in the *S. aureus* strain and absent in *S. flexneri*; the *ipaH* gene is expressed in the *S. flexneri* strain and absent in *S. aureus* (Figure 6e).

## 3. Materials and Methods

### 3.1. Materials

The tobacco packaging paper was kindly gifted from the China National Pulp and Paper Research Institute (CNPPRI, Beijing, China). *Staphylococcus aureus* and *Shigella flexneri* were collected from the China General Microbiological Culture Collection Center (CGMCC, Beijing, China). Tryptone soy broth (TSB) and nutrient broth (NB) were purchased from Beijing Land Bridge Technology Co., Ltd. (Beijing, China). Chloroauric acid (HAuCl_4_) and nitric acid (HNO_3_) were purchased from Sinopharm Chemical Reagent Co., Ltd. (Beijing, China). Sodium citrate dihydrate (C_6_H_5_Na_3_O_7_) was purchased from Alfa Aesar (Ward Hill, Massachusetts, USA). Hydrochloric acid (HCl) was purchased from Beijing Chemical Works. Crystal violet (CV) was purchased from Amresco (Solon, OH, USA). All experimental water was ultrapure water produced by the Millipore system (18.2 mΩ, Millipore). All glassware used in the experiment was immersed in aqua regia (HCl: HNO_3_ =3:1).

### 3.2. Preparation of the Au-TPP Substrates

The colloidal gold nanoparticles (Au NPs) used as an enhanced substrate were synthesized in our laboratory according to the method by Wei et al. [69]. In brief, 50 mL chloroauric acid (24.28 mM) was heated to boiling, and then 1 mL sodium citrate solution was added and heated for another 30 min. After that, the reacted solution was cooled to room temperature to obtain colloidal Au NPs. The flexible substrate of the Au-TPP was prepared by the immersion method and the centrifugation method. For the immersion method, tobacco packaging paper (square, 1 cm^2^) was soaked in Petri dishes containing colloidal Au NPs (2 mL) for different times (1 h, 6 h, 12 h, 24 h and 30 h). For the centrifugation method, tobacco packaging paper (circular paper sheets with a diameter of 2 cm) was fixed at the bottom of centrifuge tubes and 2 mL of colloidal Au NPs was added to each centrifuge tube. The centrifuge tubes were placed in a high-speed centrifuge (Z-323K, Hermle, Germany) at 10,000 rpm for 10 min, and then the supernatant was removed. The Au-TPPs were placed in a vacuum freeze dryer (LGJ-10C, Foring Technology Development Co., Ltd., Beijing, China) and oven-dried for 1 h, respectively. This operation was repeated 1–2 times in parallel to prepare the Au-TPP flexible substrates under different conditions. Finally, the Au-TPP flexible substrates were placed in vacuum-sealed storage at 4 °C.

### 3.3. Characterization of Au NPs and the Au-TPP Substrate

The position of the plasmon resonance peak of colloidal Au NPs can be detected by a UV-Vis spectrometer (UH4150, Hitachi, Japan). The scanning range was set as 200–1000 nm with a scanning speed of 600 nm/min. The morphology and size of the Au NPs were observed using a transmission electron microscope (JEM-1200EX, JEOL, Tokyo, Japan), with an accelerating voltage of 75 kV and an electric current of 40 mA. The nanostructure and surface morphology of the Au-TPP substrates were characterized using a scanning electron microscope (F50, JEOL, Tokyo, Japan).

### 3.4. SERS Detection

A portable laser Raman spectrometer (RamTracer-200, Opto Trace Technologies, Inc., Suzhou, China) equipped with a 785 nm laser, utilizing 250 mW laser power, a spectral resolution of 4 cm^−1^ and a scanning range of 250 to 1800 cm^−1^, was used to collect SERS spectra from different positions on the Au-TPP substrate.

*S. aureus* was inoculated in TSB medium, and the bacteria were placed in a 37 °C incubator and shaken at 110 rpm/min. When the optical density value of the bacterial liquid at 600 nm wavelength was 1 (OD = 1), determined using a UV-Vis spectrophotometer, the cultivation was stopped and used for standby. After centrifuging 1 mL bacterial solution at 8000 r/min for 5 min, the supernatant was discarded, and sterilized water was added and centrifuged again. *S. flexneri* was inoculated in NB medium, and the same operation was repeated as with the treatment of *S. aureus*.

Pork mince samples were purchased from a local supermarket. First, 5 g samples of pork minced meat were soaked in 10 mL of a 10 mmol/L phosphate buffer. *S. aureus* and *S. flexneri* were selected as the model pathogens, 200 μL of *S. aureus* or *S. flexneri* bacterial solution was applied to the surface of the pork, and the surface of the spiked pork was wiped with Au-TTP to collect bacteria. The number of wipes depended on the condition of pork contamination, generally 3–5 times, and the SERS test was required after each wipe.

### 3.5. PCR Technology

Pathogenic bacteria were collected from the Au-TPP substrate. Firstly, the Au-TPP substrate was rinsed with ultrapure water for about 3 min. Secondly, the ultrapure water containing colonies was centrifuged at 7000 rpm/min for 5 min. Finally, the bacterial pellet was suspended in 50 μL of ultrapure water to prepare the bacterial suspension used in the experiment.

A bacterial genomic DNA extraction kit (GD2411-01, Baierdi, Beijing, China) was used to extract the DNA of the bacterial liquid, and PCR technology was used to detect the *nuc* gene and the *ipaH* gene. The reaction conditions of the two genes were pre-denaturation at 94 °C for 5 min, denaturation at 94 °C for 45 s, annealing at 64 °C for 45 s, extension at 72 °C for 1 min and end extension at 72 °C for 10 min. The specificity of the two genes was designed by Primer software (Table 2).

## 4. Conclusions

In this study, a flexible paper-based Au substrate (Au-TPP) was prepared by a simple and fast method, showing the characteristics of high sensitivity, good uniformity, reproducibility and stability. The selected tobacco packaging paper itself has lower Raman peaks, which can reduce the background interference for the test object. Au NPs are regularly attached to the surface of paper fibers to significantly enhance the Raman signal of pathogenic bacteria. Due to the good biocompatibility of Au-TTP, rapid detection of pathogenic bacteria can be achieved, and an effective platform for screening and detection of microorganisms is also provided. When combined with portable Raman spectroscopy, this Au-TTP substrate is expected to be used as a next-generation portable biosensor for on-site pathogen detection in daily life. 

## Figures and Tables

**Table 1:** PC-LDA classification results of *S. aureus* and *S. flexneri*.

Sample	Prediction Group		Total	Sensitivity (%)	Specificity (%)
	*S. aureus*	*S. flexneri*			
*S. aureus*	34	0	34	100	100
*S. flexneri*	0	30	30	100	100

**Table 2:** The primers of *nuc* and *ipaH*.

Primer	Sequence (5′-3′)	Length
*nuc*-1	CGGTTTCGAAAGGGCAATACGCAAAGAGG	607 bp
*nuc*-2	CGATTGACCTGAATCAGCGTTGTCTTCGC	
*ipaH*-1	CGTCCGATACCGTCTCTGCACGCAATA	406 bp
*ipaH*-2	CGCCGACACGCCATAGAAACGCATTTC	

## Data Availability

Not applicable.
